# Expression of Ferritin Light Chain (FTL) Is Elevated in Glioblastoma, and FTL Silencing Inhibits Glioblastoma Cell Proliferation via the GADD45/JNK Pathway

**DOI:** 10.1371/journal.pone.0149361

**Published:** 2016-02-12

**Authors:** Tingfeng Wu, Yuntao Li, Baohui Liu, Shenqi Zhang, Liquan Wu, Xiaonan Zhu, Qianxue Chen

**Affiliations:** Department of Neurosurgery, Renmin Hospital of Wuhan University, Wuhan, Hubei province, PR China; Swedish Neuroscience Institute, UNITED STATES

## Abstract

Accumulating evidence suggests that iron-associated proteins contribute to tumor initiation and development. Ferritin light chain (FTL), a key protein in iron metabolism, is associated with the survival of glioblastoma multiforme (GBM) patients; however, the molecular mechanisms underlying this association remain largely unclear. Therefore, in the present study, we investigated the role of FTL in the pathogenesis of GBM. By using quantitative real-time RT-PCR, we found that expression of FTL was higher in patients with GBM than in those with low-grade glioma. Immunofluorescence showed that FTL was mainly localized in the nucleus of GBM cells and was closely associated with mitotic spindles. Knockdown of FTL resulted in inhibition of cell growth and activation of the GADD45A/JNK pathway in GBM cells. Immunoblotting revealed that levels of GADD45A protein decreased in GBM cells when FTL expression increased. Furthermore, transfection of GADD45A in GBM cells significantly decreased cell viability, and this effect was impeded by co-transfection of FTL. Moreover, FTL was found to localize with GADD45A in GBM cells, and a coimmunoprecipitation experiment showed that the two proteins physically interacted. Taken together, these results demonstrate a novel mechanism by which FTL regulates the growth of GBM cells via the GADD45/JNK pathway.

## Introduction

Glioblastoma multiforme (GBM) brain tumors exhibit the highest malignancy from all cancers in humans, and, due to their invasive nature, maximal surgical resection is difficult to achieve; thus, less than half of patients survive more than a year [1, 2]. Therapeutic resistance and tumor recurrence after surgery are the main causes of poor prognosis in GBM patients. In recent years, our understanding of the molecular mechanisms underlying GBM cell proliferation, angiogenesis, and immune evasion has advanced rapidly. This is particularly important because development of effective adjuvant therapy for GBM relies on thorough comprehension of its molecular pathogenesis.

Growing evidence suggests that iron-associated proteins contribute to the growth of malignant tumor cells [3]. The iron storage protein ferritin, which is essential to iron homeostasis, has predominantly been utilized in clinical medicine as a serum marker of total body iron stores; however, recent studies demonstrated that ferritin has novel functions, independent of iron storage, and showed that its expression is dysregulated in cancer [4, 5].

Ferritin is composed of functionally distinct ferritin heavy chain (FTH) and ferritin light chain (FTL) subunits; FTH possesses enzymatic activity and can oxidize ferrous iron into ferric iron, whereas FTL lacks enzymatic activity [6]. FTL levels were previously reported to positively modulate the rate of HeLa cell proliferation [7]. Interestingly, quantitative proteomic analysis of plasma from GBM patients also showed significantly elevated levels of FTL [8]. Despite these findings, the role and underlying molecular mechanisms of FTL in GBM remain largely unknown.

In this study, we assessed FTL expression in glioma samples obtained from human patients, as well as the subcellular distribution of FTL in GBM cells. Our findings suggest that FTL is 1) important to the GBM cell proliferation process, 2) a regulator of the GADD45/JNK signaling pathway, and 3) a potential regulator of GADD45A expression. Therefore, our study revealed the pivotal role played by FTL in the pathology of GBM.

## Materials and Methods

### Patient samples

The Ethics Committee of Wuhan University approved this study, and all experiments complied with the current laws of PR China. In total, 20 glioma samples were collected during May 2014 and December 2014, including both low-grade glioma (grade I, 2 cases; grade II, 8 cases) and glioblastoma multiform (grade IV, 10 cases; all defined according to the 2007 WHO classification system). All patients provided written informed consent. Samples of tumor tissue were collected during surgery, snap-frozen in liquid nitrogen, and stored until experimental use. Patients were not treated with chemotherapy or radiotherapy before surgery.

### Cells and cell culture

Three human glioblastoma-derived cancer cell lines, U251MG, A172, and U87MG, as well as a human embryonic kidney cell line, 293T (HEK 293T), were purchased directly from the Cell Bank Type Culture Collection of Chinese Academy of Sciences (Shanghai, China; the catalogue numbers were TCHu58, TCHu171, TCHu138, and GNHu17). All cell lines were maintained in Dulbecco’s modified Eagle’s medium (Gibco, Invitrogen, Carlsbad, CA, USA) with 10% fetal bovine serum (Gibco) and 1% penicillin-streptomycin (Sigma-Aldrich) at 37°C under a humidified atmosphere of 5% CO_2_. The working concentration of MG132 used in cell experiments was 10 μM.

### Real-time quantitative reverse transcription polymerase chain reaction (RT-PCR)

Real-time quantitative RT-PCR was used to analyze mRNA expression in patient samples and cell lines. Total RNA was extracted using TRIzol reagent (Invitrogen), and cDNA was prepared from 2–6 μg of total RNA by using the PrimeScript RT Reagent Kit with gDNA Eraser (TaKaRa, Tokyo, Japan) and random hexamer primers. To detect FTL in 2 μl of cDNA, real-time PCR was employed with SYBR Green II Mixture (TaKaRa) according to the manufacturer’s protocol. The specific primer pairs were as follows: FTL primer (forward primer, 5´-CAGCCTGGTCAATTTGTACCT-3´; reverse primer, 5´-GCCAATTCGCGGAAGAAGTG-3´), GAPDH (internal control gene) primer (forward primer, 5´-ACAACTTTGGTATCGTGGAAGG-3´; reverse primer, 5´-GCCATCACGCCACAGTTTC-3´). Real-time PCR comprised an initial denaturation at 95°C for 30 s, and then 40 cycles at 95°C for 5 s and 60°C for 34 s. The data were analyzed using ABI Prism 7500 SDS v1.4 software (Applied Biosystems, Foster City, CA, USA). For all specimens, procedures were performed in triplicate and the resulting data were averaged.

### Plasmid construction and transfection

Human FTL (GenBank ID: NM000146) full-length cDNA was subcloned into a pcDNA3-myc vector to generate the myc-FTL construct. Human Gadd45a (GenBank ID: NM000146) full length cDNA was subcloned into a pEnter-flag vector to generate the flag-Gadd45a construct. All cell lines in this study were transfected with plasmids by using Lipofectamine 2000 (Invitrogen) or Attractene transfection reagent (Qiagen, Valencia, CA, USA) according to the manufacturers’ protocols.

### FTL-targeted small-interfering RNA (si-RNA) transfection

Glioma cells were seeded to 50–75% confluency in a 6-well plate. After 24 h, si-RNA transfections were conducted in serum-containing conditions using HyperFect (Qiagen) according to the manufacturer’s instructions. Cells were harvested 72 h after transfection, and FTL knockdown was verified by using both western blotting and RT-PCR. Three distinct si-RNA sequences and non-specific si-RNA (as a negative control) were used. The sequences of the si-RNA against human FTL mRNA (designed and synthesized by Guangzhou RiboBio Co., Ltd.) were as follows: si-FTL-1, sense 5´-CCUGGAGACUCACUUCCUA dTdT-3´ and antisense 5´-UAGGAAGUGAGUCUCCAGG TdTd-3´; si-FTL-2, sense 5´- GGCGAGUAUCUCUUCGAAA dTdT-3´ and antisense 5´- UUUCGAAGAGAUACUCGCC TdTd-3´; si-FTL-3, sense 5´- CCAGAUUCGUCAGAAUUAU dTdT-3´ and antisense 5´- AUAAUUCUGACGAAUCUGG TdTd-3´. All si-RNAs were used at a final concentration of 20 nM.

### Cell proliferation assay

U251MG, A172, and U87MG cell growth was measured 24, 48, and 72 h after transfection with FTL si-RNA by using the Cell Counting Kit-8 (CCK-8; Dojindo Molecular Technologies, Inc., Rockville, MD, USA), according to the manufacturer’s protocol. On average, six replicates for each time point were statistically analyzed.

### Western blot analysis

Glioma cells lysates were prepared by sonicating cells briefly in a modified RIPA buffer (0.1% SDS, 50 mM Tris-HCl, pH 7.5, 150 mM NaCl, 0.1% sodium deoxycholate, 1% Nonidet P-40 in Milli-Q) with proteinase and phosphatase inhibitors. Protein was quantified by using the BCA protein assay (TaKaRa). Lysates were separated by 4–15% SDS-PAGE and transferred to a PVDF membrane (Millipore, Bedford, USA). Blocking and incubation with antibodies was performed in PBS containing 0.05% Tween-20 with 1% bovine serum albumin. The primary antibodies used in this study recognized the following proteins: human FTL (Abcam, Cambridge, MA, USA: Ab109019; 1:1000), PCNA (Cell Signaling Technology, Danvers, MA, USA: 13110; 1:1000), topoisomerase II-alpha (Abcam: Ab2987; 1:100), GAPDH (Santa Cruz Biotechnology, CA, USA: sc-25778; 1:1000), p-Histone H3 (Ser10) (Santa Cruz Biotechnology: sc-8656-R; 1:1000), Histone H3 (Cell Signaling Technology: 4499; 1:1000), GADD45a (Santa Cruz Biotechnology: sc-6850; 1:200), Cyclin D1 (Cell Signaling Technology: 2978; 1:1000), c-Myc (Santa Cruz Biotechnology: sc-40; 1:1000), PDGFR-β (Santa Cruz Biotechnology: sc-432; 1:1000), p-JNK (Santa Cruz Biotechnology: sc-6254; 1:1000), JNK (Santa Cruz Biotechnology: sc-7345; 1:1000), p-Histone H2A.X (Ser139) (Cell Signaling Technology: 9718; 1:1000), and Histone H2A (Cell Signaling Technology: 12349; 1:1000). Goat anti-Rabbit and Goat anti-Mouse infrared dye secondary antibodies (800CW) were purchased from LI-COR Biosciences (Lincoln, NE, USA; 1:10000). Proteins were visualized with the Odyssey Bioanalyzer (LI-COR). Western blots were repeated three times for all samples.

### Co-immunoprecipitation experiments

HEK 293T cells in a 10-cm Petri dish were transfected with myc-FTL, flag-Gadd45a, and their control expression vectors in different combinations using the Attractene Transfection Reagent (Qiagen). Forty-eight hours after transfection, cells were rinsed twice with PBS and lysed in 1 ml lysis buffer (25 mM Tris-HCl, pH 7.5, 150 mM NaCl) containing 0.5% Nonidet P-40 with proteinase and phosphatase inhibitors. The cell lysates were briefly centrifuged to remove cell debris, and the supernatant was separated into three aliquots (300 μl per aliquot). The first aliquot was incubated with 10 μl of mouse anti-myc monoclonal antibody (Santa Cruz Biotechnology: sc-40), the second aliquot was incubated with 2 μl of mouse anti-flag monoclonal antibody (MBL, Nagoya, Japan: M185-3L), and the third aliquot was incubated with 2 μl of mouse monoclonal anti-IgG antibody (Abcam: Ab18413). After incubation at 4°C overnight, the immune complex was precipitated with 20 μl Protein G Plus-Agarose (Santa Cruz Biotechnology: sc-2002). The immunoprecipitates were washed four times with lysis buffer, and then subjected to electrophoresis. The antibodies used for western blotting were rabbit anti-myc antibody (Santa Cruz Biotechnology: sc-789) and rabbit anti-flag antibody (Sigma-Aldrich: F7425).

### Immunocytochemistry and immunohistochemistry

Glioma cells were fixed with 4% paraformaldehyde, permeabilized with 0.1% Triton X-100 in PBS, and then blocked with 1% donkey serum. After incubation overnight with the primary antibodies, rabbit anti-FTL (Abcam: Ab109019; 1:250) and mouse anti-GADD45a (Santa Cruz Biotechnology: sc-6850; 1:200), the cells were washed with 1% donkey serum. Secondary antibodies, goat anti-rabbit-Alexa 488 (Invitrogen: A-11008; 1:300) and donkey anti-mouse-Alexa 594 (Invitrogen: A-21203; 1:300), were then added to the cells for 60 min. Cell nuclei were counterstained with DAPI (Invitrogen: D1306). For double staining, mouse antibodies were first added for 60 min, and then the rabbit antibody was added for 60 min.

### Statistical analyses

FTL mRNA expression, 2^-ΔΔCT^, as measured using RT-PCR in the different grades of glioma tissue, was compared using unpaired t-tests. For the cell proliferation assay, the differences between the two groups were analyzed using the Student’s t-test. The western blotting data were analyzed with analysis of variance (ANOVA). Data are presented as mean ± standard deviation. All statistical analyses were conducted in SAS (Version 9.2; SAS Institute, Cary, NC, USA). P < 0.05 was considered statistically significant.

## Results

### FTL expression was higher in glioblastoma than in low-grade glioma, and decreased expression of FTL correlated with increased survival in glioblastoma patients

**Fig 1:**
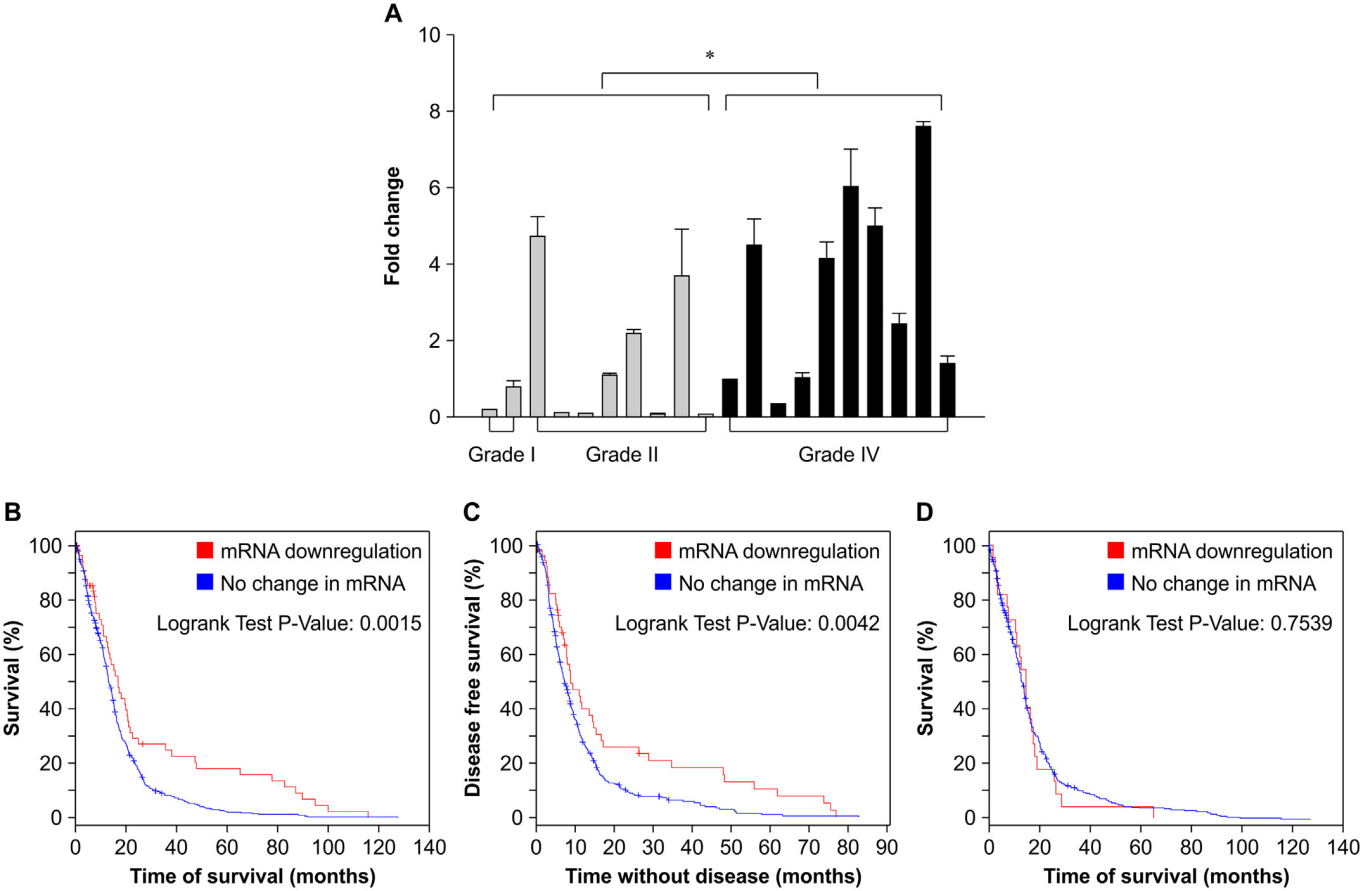
Ferritin light chain expression in glioma and its correlation with survival in glioblastoma multiforme patients. (A) Ferritin light chain (FTL) expression levels were measured using real-time quantitative RT-PCR. Expression of FTL was significantly higher in the glioblastoma multiforme (GBM) group (grade IV) compared with the low-grade glioma group (grades I and II) (*P = 0.04). Data represent the mean ± standard deviation of triplicate experiments. (B and C) In silico analyses of overall survival and disease free survival of GBM patients according to their expression levels of FTL mRNA. Data were obtained from cBioPortal datasets [9, 10]. (D) In silico analysis showing overall survival of GBM patients according to their expression levels of FTH1 mRNA. Data were also obtained from the cBioPortal datasets [9, 10].

For FTL mRNA expression, the geometric mean in the low-grade glioma group was 0.4, whereas it was 2.3 in glioblastoma multiform group. FTL expression levels were significantly higher in the glioblastoma multiform group compared with levels in the low-grade glioma group (P = 0.04; Fig 1A). In silico analysis of the public U133 microarray datasets (TCGA), which were obtained from the cBioPortal for Cancer Genomics (http://cbioportal.org), revealed a trend for higher overall survival (logrank P = 0.001) and higher disease-free survival (logrank P = 0.004) in patients that exhibited downregulation of FTL mRNA (>2-fold decrease) (Fig 1B and 1C). However, in silico analysis showed no significant difference in the overall survival (logrank P = 0.75) of patients that exhibited downregulation of FTH1 (>2-fold decrease) and patients that exhibited no change in FTH1 mRNA (Fig 1D).

### FTL was mainly localized in the nucleus of GBM cells and involved in cell mitosis

**Fig 2:**
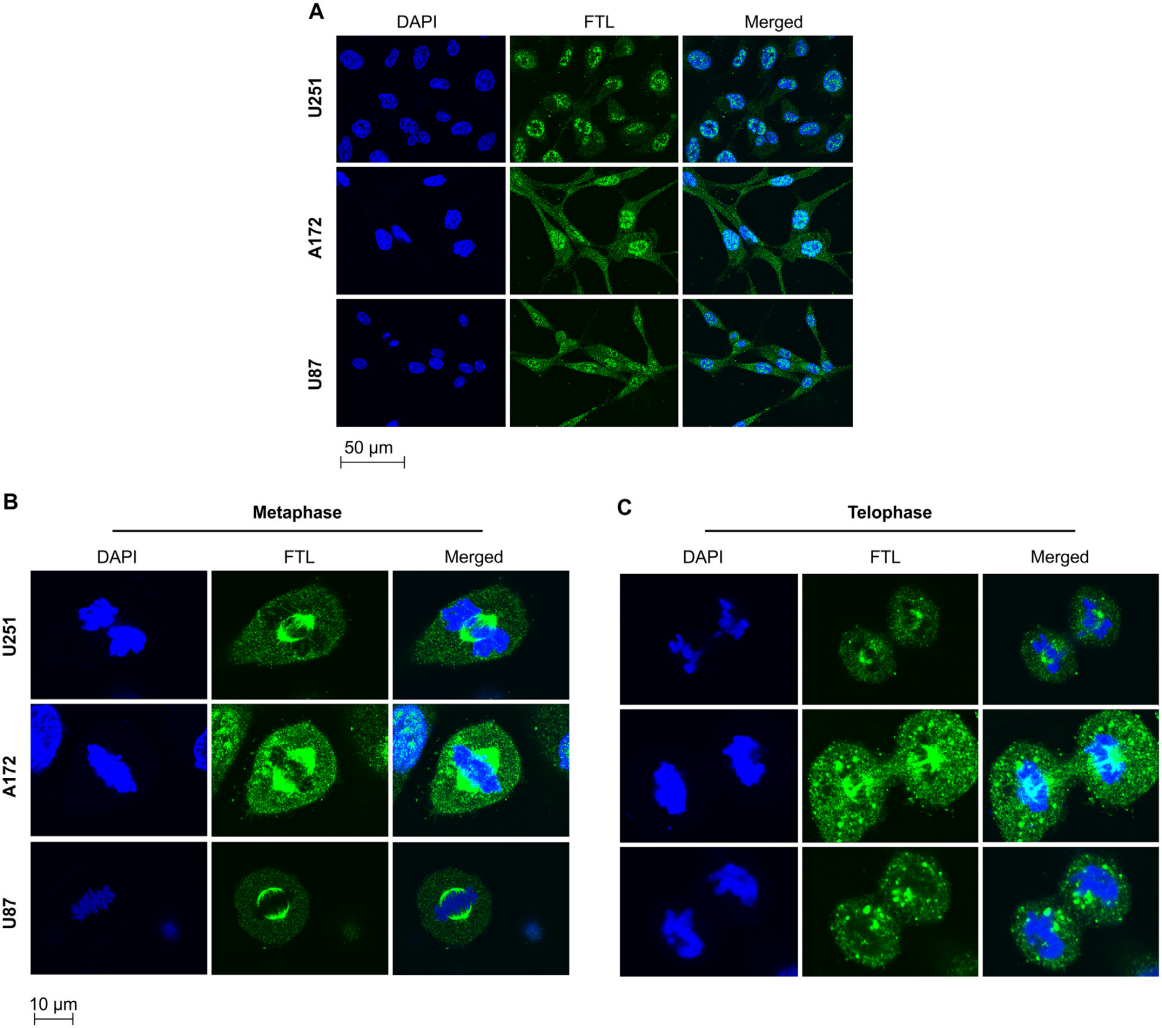
Subcellular localization of ferritin light chain in glioblastoma multiforme cell lines during cell cycle phases. (A–C) U251, A172, and U87 glioblastoma multiforme cells in different phases of the cell cycle were immunostained with ferritin light chain (FTL) antibodies (green). Nuclei were stained with DAPI (blue).

We applied immunofluorescence to identify the subcellular distribution of FTL in U251, A172, and U87 GBM cells. FTL was mainly localized in the cell nucleus at interphase (Fig 2A). During cell mitosis, FTL was present in mitotic spindles at metaphase and distributed equally in the dividing cell nucleus at telophase (Fig 2B and 2C).

### si-RNA-mediated knockdown of FTL in three GBM cell lines led to inhibition of growth

**Fig 3:**
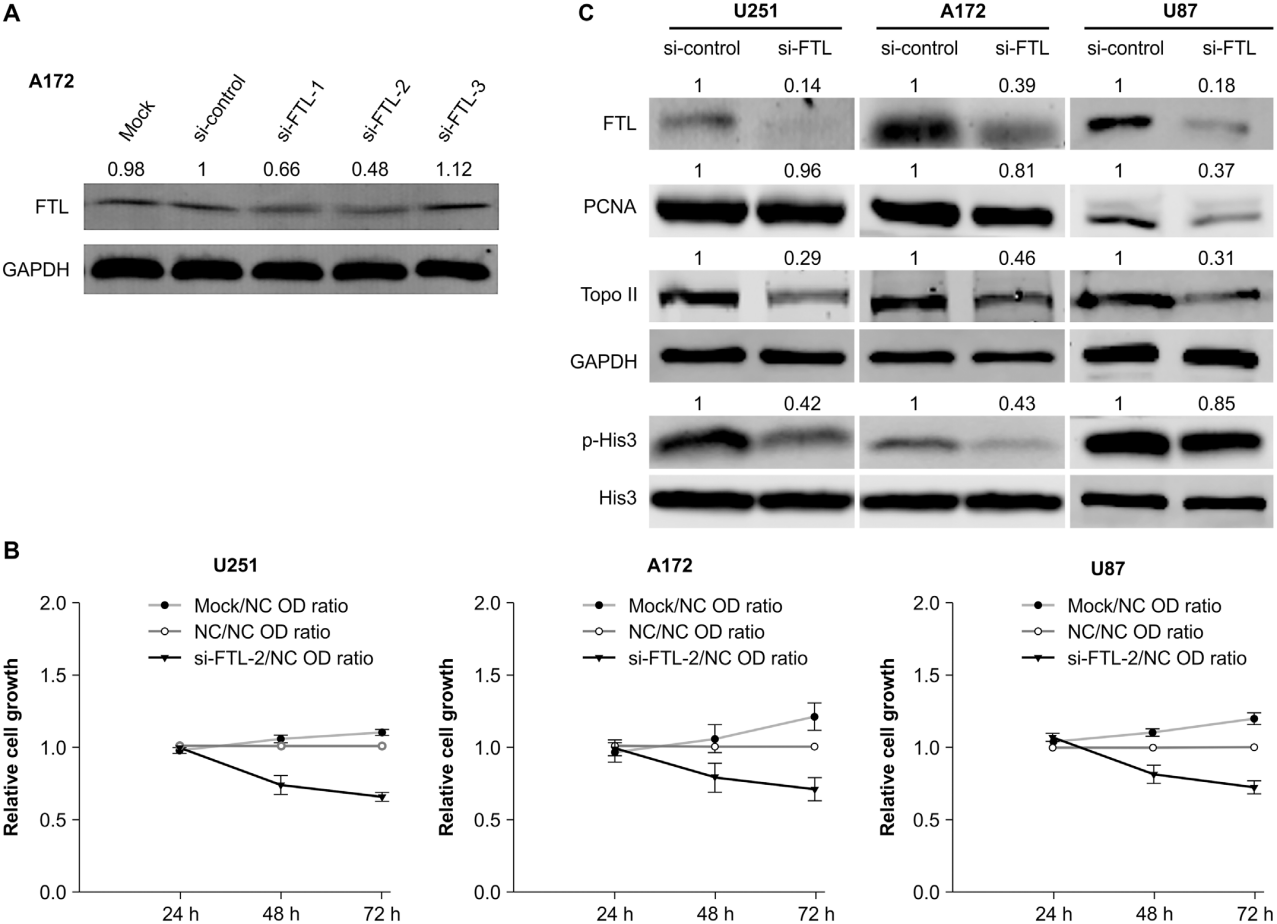
si-RNA-mediated knockdown of ferritin light chain in glioblastoma multiforme cell lines inhibits cell growth. (A) Protein blots of samples harvested 72 h after silencing ferritin light chain (FTL) in A172 glioblastoma multiforme (GBM) cells with three respective si-RNAs: si-FTL-1, si-FTL-2, and si-FTL-3. Of these, si-FTL-2 showed the best interference efficiency (si-control group vs. si-FTL-2 group: P < 0.01). “Mock” denotes treatment with transfection reagent only. “si-Control” denotes transfection with scrambled si-RNA as a negative control. GAPDH was used as an internal loading control. The number at the top of each band represents the average densitometric value normalized against that of GAPDH. (B–D) Results of Cell Counting Kit-8 (CCK-8) assays 24, 48, and 72 h after transfection of GBM cells with mock, si-FTL-2, or scrambled control (negative control: NC) si-RNAs. The data points represent the OD ratio of Mock/NC, NC/NC, si-FTL-2/NC at different time points. Data represent the mean ± standard deviation of six replicates per time point. (E) Immunoblot analyses of U251, A172, and U87 GBM cells. Downregulation of FTL resulted in reduced expression of topoisomerase II-alpha (Topo II) and p-Histone H3 (Ser10) (p-His3) in all cell lines (P < 0.01). PCNA expression also decreased in A172 and U87 cells (P < 0.01), but not in U251 cells (p = 0.04). GAPDH was used as an internal loading control. The number at the top of each band represents the average densitometric value from three experiments, normalized against that of GAPDH (FTL, Topo II and PCNA) or His3 (p-His3).

We silenced FTL using three distinct si-RNAs: si-FTL-1, si-FTL-2, and si-FTL-3. In western blotting analysis, si-FTL-2 (targeting sequence: 5´- GGCGAGTATCTCTTCGAAA-3´) showed the best interference efficiency in A172 GBM cells (si-control group vs. si-FTL-2 group: P<0.01) (Fig 3A); therefore, this sequence was selected to knockdown the endogenous FTL in GBM cells. A CCK-8 assay was used to evaluate U251, A172, and U87 GBM cell growth 24, 48, and 72 h after si-RNA transfection. Cell growth significantly decreased in all cells transfected with si-FTL-2 relative to those transfected with scrambled si-RNA (negative control) at 48 h (P < 0.01) and at 72 h (P < 0.01) (Fig 3B–3D). Immunoblotting revealed that expression of the mitosis marker, p-Histone H3 (Ser10), and the proliferation marker, topoisomerase II-alpha, decreased in all cell lines following knockdown of FTL (P < 0.01), whereas PCNA expression decreased in A172 and U87 (P < 0.01), but not U251 (P = 0.04), cells (Fig 3E).

### The GADD45A/JNK pathway was activated by knockdown of FTL in GBM cells

**Fig 4:**
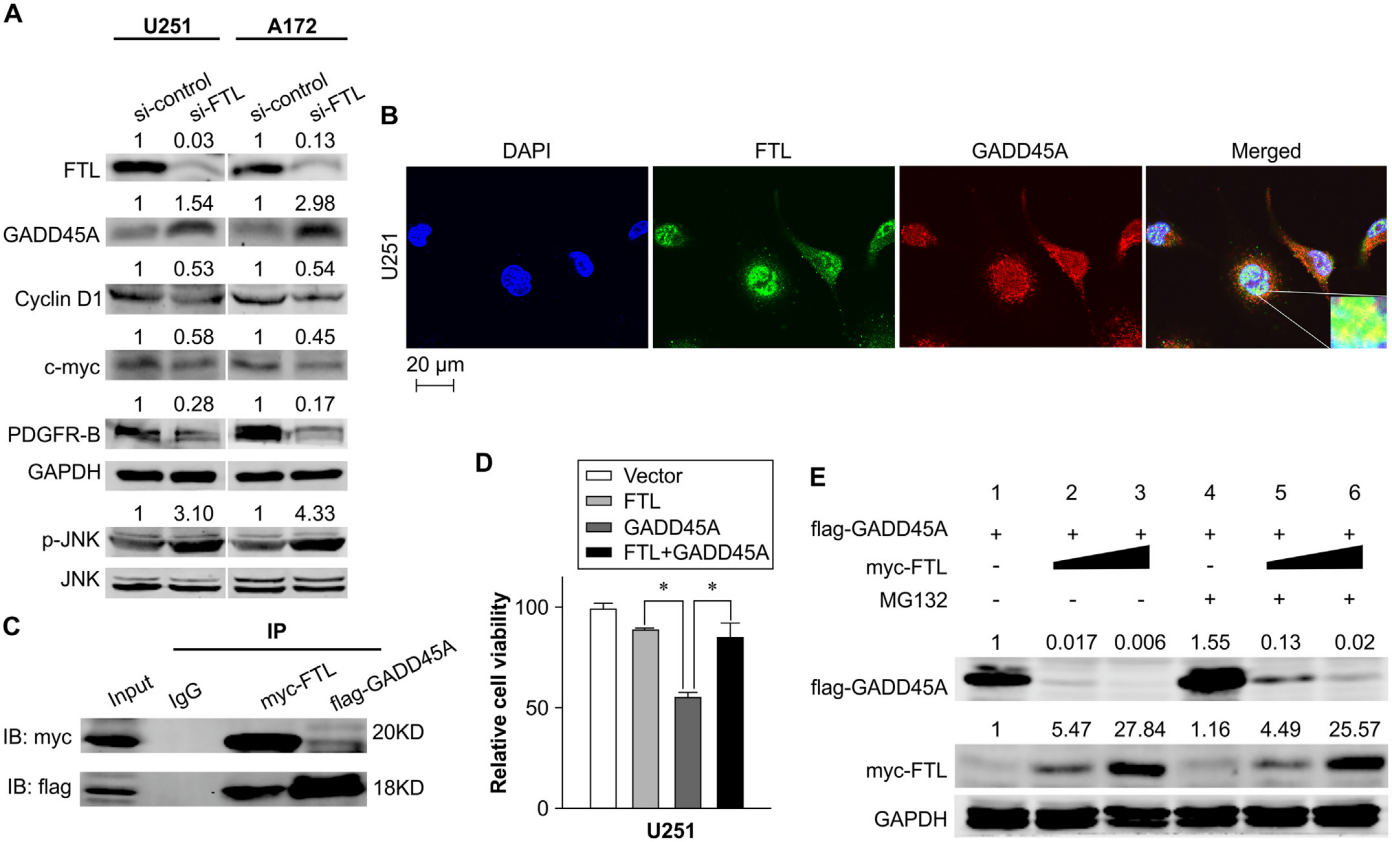
Ferritin light chain regulates the GADD45A/JNK pathway and interacts with GADD45A in glioblastoma multiforme cells. (A) Immunoblot analyses showing the effects of ferritin light chain (FTL) knockdown in U251 and A172 cells. Downregulation of FTL activated the GADD45A/JNK pathway in both cell lines, which was indicated by elevated levels of GADD45A, activation of JNKs (P < 0.01). Expression of Cyclin D1, c-myc, and PDGFR-β was also observed. The number at the top of each band represents the average densitometric value from three experiments, normalized against that of GAPDH. (B) U251 glioblastoma multiforme GBM cells were co-immunostained with FTL antibodies (green) and GADD45A antibodies (red), and nuclei were stained with DAPI (blue). (C) Co-immunoprecipitation and immunoblotting analyses confirmed the interaction between FTL and GADD45A proteins. (D) Results of a Cell Counting Kit-8 (CCK8) assay where U251 cells were transfected with the indicated plasmids for 48 h. Cell viability, relative to cells transfected with a control plasmid (vector), is indicated. (E) GADD45A protein levels in FTL expression vector-transfected 293T cell lysates as measured by immunoblotting. Along with elevated FTL protein levels, a decrease in GADD45A protein levels was observed; this effect was attenuated by treatment with a proteasome inhibitor, MG132. The number at the top of each band represents the average densitometric value from three experiments, normalized against that of GAPDH.

Evidence from a protein-protein interaction database [11, 12] shows that FTL interacts with proteins that play important roles in the PIK3/AKT, GADD45/JNK, and TGF-β signaling pathways and cell cycle regulation. Our experiments revealed that FTL knockdown in U251 and A172 GBM cells resulted in elevated levels of GADD45A and activation of JNKs (P < 0.01)(Fig 4A). In addition, FTL knockdown substantially decreased the expression of the Wnt target genes Cyclin D1 and c-myc (P < 0.01) (Fig 4A). Furthermore, we found that inhibition of FTL significantly decreased expression of PDGFR-β (P < 0.01) (Fig 4A).

### FTL localized with GADD45A in the nucleus of GBM cells and physically interacted with GADD45A

Immunofluorescence analyses showed that FTL localized with GADD45A in the nucleus of U251 GBM cells (Fig 4B). Following co-transfection of myc-FTL and flag-GADD45A in HEK 293T cells, and the subsequent mutual coimmunoprecipitation, FTL was also found to physically interact with GADD45A (Fig 4C).

### FTL sustains GBM cell viability by inhibition of GADD45A expression

Analysis of immunoblotting revealed that increased expression of FTL protein inhibited the expression of GADD45A protein, and this process was significantly attenuated by treatment with MG132, a proteasome inhibitor (Fig 4E). Furthermore, transfection of GADD45A in GBM cells significantly decreased cell viability (P < 0.01); however, this decrease was impeded by co-transfection of FTL (P < 0.01) (Fig 4D).

## Discussion

It is generally accepted that ferritin is the primary iron storage protein, but recent studies have demonstrated that it may also be pivotal to cell proliferation, angiogenesis, and immunosuppression [6]. Tumor ferritins are composed of different ratios of the two functionally distinct ferritin subunits, FTH and FTL [12, 13]. Silencing FTH can effectively sensitize GBM cells to the DNA-alkylating agent carmustine (also known as BCNU), which is commonly used as a chemotherapy drug [14]. However, by using the cBioPortal for Cancer Genomics [9, 10], we found that FTL rather than FTH was closely associated with survival of GBM patients. FTL has previously been detected in the plasma of GBM patients at significantly elevated levels [12, 15]. Given these findings, in the present study, we investigated the role of FTL in the molecular pathology of GBM.

Our results showed that FTL expression was higher in GBM patients than in those with low-grade glioma, and that overall survival and disease-free survival was higher in patients with downregulated FTL mRNA. These findings suggest that FTL has potential prognostic value for GBM patients.

We also found that FTL was mainly localized in the nucleus of GBM cells, and, surprisingly, that it was present in mitotic spindles at metaphase and distributed equally in the dividing cell nucleus at telophase. Similarly, in previous studies, genes that are closely associated with the mitotic spindle, such as survivin and TPX2, have been identified as markers for the diagnosis and prognosis of cancer [16, 17]. Additionally, Kittler et al. [18] found that FTL was required for HeLa cell division. Given this evidence, it is reasonable to assume that FTL could disturb the mitotic process of GBM cells; therefore, we also investigated the function of FTL in GBM cell growth. Results showed that si-RNA-mediated FTL silencing significantly inhibited GBM cell growth. This was confirmed by immunoblotting, whereby significant downregulation of the mitosis marker p-Histone H3 (Ser10) and the proliferation marker topoisomerase II-alpha was also observed. Thus, our findings suggest that FTL is as an important player in the GBM cell growth process.

We used multiple methods, including bioinformatics, to identify the key genes related to FTL-mediated GBM cell growth. We found that knockdown of FTL in GBM cells resulted in elevation of GADD45A levels and activation of JNKs. Previous investigations have shown that increased GADD45A expression correlates with increased survival rate for GBM patients [19]. High levels of GADD45A are also correlated with impaired cell proliferation [20]. Furthermore, cancer cells with GADD45A knockdown can evade the apoptotic pathway [21], and GADD45A elicits its function through activation of JNK and p38 kinases [22, 23]. We therefore speculate that the effects observed following FTL knockdown in our study rely on the GADD45/JNK pathway. Our immunoblotting analysis also revealed that GADD45A protein levels decreased when GBM cells expressed higher levels of FTL. Additionally, transfection of GADD45A led to significantly decreased GBM cell viability, which was hindered by co-transfection of FTL. Taken together, our findings provide evidence for the role of FTL as a regulator in the GADD45/JNK pathway, which is pivotal to GBM cell growth.

Our results showed that FTL localized with GADD45A in the nucleus of GBM cells, and that these proteins physically interacted. The protein–protein interaction of FTL and GADD45A was previously indicated in a PPI network study [24], and our study provides further supporting evidence for this physical interaction. Structural studies have demonstrated that homo- and hetero-dimerization of GADD45 isoforms are essential for the activation of these proteins and their anti-proliferative functions [25]. Based on our study, we suspect that FTL could interfere with the dimerization of GADD45A and thereby contribute to its degradation; however, further research will be required to comprehensively evaluate this process.

We found that FTL knockdown substantially decreased the expression of two Wnt target genes, Cyclin D1 and c-myc. Previous studies indicate that GADD45A induction can inhibit the activation of Wnt signaling [26]. Moreover, because Wnt signaling is strongly activated in mitosis [27], our results provide a possible explanation for the role of FTL in mitosis. In addition, we found that PDGFR-β expression decreased after FTL knockdown in GBM cells. Because the overexpression and excessive signaling of PDGFR-β has been detected in GBM, and since PDGFR-β represents a therapeutic target in GBM [28, 29], our results suggest a potential role for FTL in GBM therapy.
